# Surveillance Metrics of SARS-CoV-2 Transmission in Central Asia: Longitudinal Trend Analysis

**DOI:** 10.2196/25799

**Published:** 2021-02-03

**Authors:** Lori Ann Post, Elana T Benishay, Charles B Moss, Robert Leo Murphy, Chad J Achenbach, Michael G Ison, Danielle Resnick, Lauren Nadya Singh, Janine White, Azraa S Chaudhury, Michael J Boctor, Sarah B Welch, James Francis Oehmke

**Affiliations:** 1 Buehler Center for Health Policy and Economics Feinberg School of Medicine Northwestern University Chicago, IL United States; 2 Feinberg School of Medicine Northwestern University Chicago, IL United States; 3 Institute of Food and Agricultural Sciences University of Florida Gainsville, FL United States; 4 Institute for Global Health Feinberg School of Medicine Northwestern University Chicago, IL United States; 5 Divison of Infectious Disease Feinberg School of Medicine Northwestern University Chicago, IL United States; 6 International Food Policy Research Institute Washington, DC United States

**Keywords:** SARS-CoV-2 surveillance, second wave, wave two, global COVID-19 surveillance, Central Asia public health surveillance, Central Asia COVID-19, Central Asia surveillance metrics, dynamic panel data, generalized method of moments, Central Asia econometrics, Central Asia SARS-CoV-2, Central Asia COVID-19 surveillance system, Central Asia COVID-19 transmission speed, Central Asia COVID transmission acceleration, COVID-19 transmission deceleration, COVID-19 transmission jerk, COVID-19 7-day lag, SARS-CoV-2, Arellano-Bond estimator, generalized method of moments, GMM, Armenia, Azerbaijan, Cyprus, Faeroe Islands, Georgia, Gibraltar, Kazakhstan, Kosovo, Kyrgyzstan, Macedonia, Russia, Tajikistan Turkey, Turkmenistan, Uzbekistan, COVID-19, surveillance, longitudinal, trend, trend analysis, monitoring, public health, infectious disease, transmission, risk, management, policy, prevention

## Abstract

**Background:**

SARS-CoV-2, the virus that caused the global COVID-19 pandemic, has severely impacted Central Asia; in spring 2020, high numbers of cases and deaths were reported in this region. The second wave of the COVID-19 pandemic is currently breaching the borders of Central Asia. Public health surveillance is necessary to inform policy and guide leaders; however, existing surveillance explains past transmissions while obscuring shifts in the pandemic, increases in infection rates, and the persistence of the transmission of COVID-19.

**Objective:**

The goal of this study is to provide enhanced surveillance metrics for SARS-CoV-2 transmission that account for weekly shifts in the pandemic, including speed, acceleration, jerk, and persistence, to better understand the risk of explosive growth in each country and which countries are managing the pandemic successfully.

**Methods:**

Using a longitudinal trend analysis study design, we extracted 60 days of COVID-19–related data from public health registries. We used an empirical difference equation to measure the daily number of cases in the Central Asia region as a function of the prior number of cases, level of testing, and weekly shift variables based on a dynamic panel model that was estimated using the generalized method of moments approach by implementing the Arellano-Bond estimator in R.

**Results:**

COVID-19 transmission rates were tracked for the weeks of September 30 to October 6 and October 7-13, 2020, in Central Asia. The region averaged 11,730 new cases per day for the first week and 14,514 for the second week. Infection rates increased across the region from 4.74 per 100,000 persons to 5.66. Russia and Turkey had the highest 7-day moving averages in the region, with 9836 and 1469, respectively, for the week of October 6 and 12,501 and 1603, respectively, for the week of October 13. Russia has the fourth highest speed in the region and continues to have positive acceleration, driving the negative trend for the entire region as the largest country by population. Armenia is experiencing explosive growth of COVID-19; its infection rate of 13.73 for the week of October 6 quickly jumped to 25.19, the highest in the region, the following week. The region overall is experiencing increases in its 7-day moving average of new cases, infection, rate, and speed, with continued positive acceleration and no sign of a reversal in sight.

**Conclusions:**

The rapidly evolving COVID-19 pandemic requires novel dynamic surveillance metrics in addition to static metrics to effectively analyze the pandemic trajectory and control spread. Policy makers need to know the magnitude of transmission rates, how quickly they are accelerating, and how previous cases are impacting current caseload due to a lag effect. These metrics applied to Central Asia suggest that the region is trending negatively, primarily due to minimal restrictions in Russia.

## Introduction

### Background

On December 29, 2019, 4 cases of “pneumonia of unknown etiology” were reported in Wuhan, Hubei Province, China [1]. What began as 4 isolated cases escalated into a global pandemic of SARS-CoV-2, the virus that causes COVID-19. To date, the caseload has reached 68,645,081** **confirmed cases, and 1,564,496 deaths have been confirmed globally [2]. Nations have been greatly impacted by the pandemic, which has resulted in significant morbidity and mortality, food insecurity, and an economic recession that has not bottomed out. Global leaders are struggling to balance disease control with salvaging their plummeting economies in the face of a global pandemic [3]. This study aims to examine where and when SARS-CoV-2 was transmitted in Central Asia within the context of a global pandemic by delving into the environmental, sociocultural, and public health characteristics of COVID-19. For the purposes of this study, we use the World Bank’s definition of Central Asia, which includes Armenia, Azerbaijan, Cyprus, Faeroe Islands, Georgia, Gibraltar, Kazakhstan, Kosovo, Kyrgyzstan, Macedonia, Russia, Tajikistan, Turkey, Turkmenistan, and Uzbekistan [4].

### History of Central Asia

Central Asia is largely composed of nation states that are former Soviet Union member countries. The Union of Soviet Socialist Republics (USSR) was dissolved in 1991 after controlling the region for 68 years, following a coup d’état during President Gorbachev’s administration. The former Soviet Union left a lasting legacy in the former national republics [5]. The USSR shifted from a centralized government and systems to independent states. Many essential institutions, such as national currency systems and military forces, were developed from the ground up. Many of these fledgling nations failed to achieve democracies, which led to more chaos and conflict. The “colored revolutions” from 1999-2005, a series of mass protests and riots, lead to the overthrow of the semiauthoritarian regimes in Serbia, Georgia, Ukraine, and Kyrgyzstan [6].

Public health systems in these new nations in Central Asia faced many challenges, including endemic infectious diseases [7] such as tuberculosis, HIV, and substance use disorders [8,9]. Alcohol poisoning is relatively common in the former USSR [10], contributing to low life expectancy in Russia [11,12]. Central Asia has one of the highest prevalence rates of tobacco misuse [13]. Misuses of alcohol, tobacco, and other substances represent both direct and indirect COVID-19 risk factors. Drinking alcohol, smoking, and misusing substances increases a person’s risk of being infected with SARS-CoV-2 and having worse outcomes if infected [14-17]. Moreover, persons who are self-isolating are at higher risk of substance use disorders [18-20]. Substance use disorders also contribute to increases chronic disease [21-23]. During past epidemics, reduced access to medical care for individuals with serious illness, such as HIV or tuberculosis, resulted in more deaths from complications due to a lack of health care resources [24,25].

### Food and Water Security

Progress in food security [26] has stalled in Central Asia in recent years, with growth in malnourished and undernourished populations [27]. Malnourishment is a function of poverty [28,29].

Food insecurity is linked to environmental conditions caused by overuse of the Aral Sea, which has been depleted by 90% since 1960 to irrigate large areas of land [30,31]. Safe water is not available for 22 million people (31% of the population) throughout Kazakhstan, Kyrgyzstan, Uzbekistan, Tajikistan, and Turkmenistan. The majority of the affected individuals live in rural areas, where there are limited sewer connections and septic tanks [32]. People living near the highly polluted Aral Sea have higher levels of tuberculosis, anemia, and cancer, and they may be at higher risk of debilitating SARS-CoV-2 infection [33].

Beyond access to food, obesity is becoming more pervasive [34] as Central Asian foods mirror the “Western diet” of high fat and low grains [26]. Traditional dishes have high contents of sodium, likely due to the “Silk Road” pattern in which countries along the former Silk Road, a trade route through Central Asia established in the second century BC [35,36], use large quantities of salt for food preservation [37]. Obesity also contributes to chronic metabolic diseases and is associated with worse outcomes in those infected with SARS-CoV-2 [38].

### Current Politics

Because Kazakhstan and China share a border, preventative measures in Kazakhstan were established as early as January 6, 2020, enforcing increased border sanitation and monitoring arrivals from China [39]. The first cases of COVID-19 in Kazakhstan, discovered in people arriving from Germany and Italy, were recorded on March 13. A state of emergency was announced on March 16; schools converted to remote learning, and quarantines were established in some areas as early as March 19 [39].

Many countries in the region have had disruptions in the labor market. The oil economies in Kazakhstan and Azerbaijan have been negatively impacted [40]. Tajikistan has experienced rising unemployment [29], and the poverty rate has risen across Central Asia [41-46].

State governments have attempted to minimize the impact of the virus; complaints about lack of adequate personal protective equipment in Russia were suppressed [47], medical authorities who gave public health advice in Turkey were criminally investigated [48], and the oppressive Turkmenistan government denied the existence of even a single case of SARS-CoV-2 [49].

In the electoral authoritarian regime of Azerbaijan, lockdown was established immediately following the first confirmed case of SARS-CoV-2 on February 28. The capital of Azerbaijan instated highly restrictive rules and shut down its border with the Islamic Republic of Iran, where cases were spreading quickly, the next day [50].

In Azerbaijan, violations of the quarantine mandate were punishable by fines, custodial restraint, and prison time [51]. Severe rules were briefly in place; people aged over 65 years were prohibited from leaving their homes [50], and citizens were required to send an SMS text message to a government telephone number to request permission to leave their home for up to three hours. The government inactivated the cellular service of political rivals so that they could not request permission to leave their residences and thus were confined to their homes [51]. The pandemic has been exploited as a means to restrict individual human rights in other countries as well; government access to private cell phone data was allowed in Armenia [52], and protests were restricted in Russia [53]. In both Uzbekistan and Tajikistan [54], penalties were introduced for the spread of false information about the virus through the media.

While Azerbaijan initially slowed the spread of the virus [55] and relaxed restrictions in May, the strict quarantine regime was reinstated in four major cities on June 21, 2020, after a surge in cases.

Shocking the international community, Russian President Vladimir Putin announced on August 11 that their country’s health regulator was the first in the world to approve a SARS-CoV-2 vaccine for mass use [56]. This approval has been criticized as unsafe because the standard phase III clinical trials for new drugs were not completed at the time of his announcement [57]. While Russia’s first approved vaccine, known as “Sputnik V,” was still undergoing phase III trials, on October 14, President Putin announced the approval of a second SARS-CoV-2 vaccine [58]. Without adequate testing, we do not know the possible detrimental side effects of these vaccines, which may undermine attempts by the international community to provide safe immunization [56].

**Figure 1:**
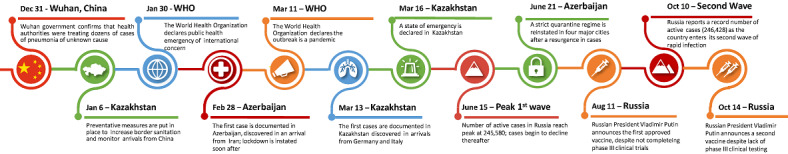
Timeline of the COVID-19 pandemic in Central Asia.

Other than vaccine efforts, Russia has not implemented significant public health measures. During the first wave of infections in March 2020, no financial support was given to small or medium-sized businesses despite instructions for employees to stay home on paid leave [59]. However, in mid-April, it was announced that over 100 billion rubles (US $1.325 billion) would be allocated to support small and medium-sized businesses [54]. The number of active cases in Russia reached a peak of 245,580 on June 15 and then began to decline [60]. With the second wave of infections in October after loosening restrictions, Russia saw a spike in cases and reported the fourth highest number of SARS-CoV-2 cases worldwide, behind the United States, India, and Brazil [61]. Russia reported a record number of 29,039 active cases on December 6 [60]. Despite record numbers of daily cases, with 26,097 new cases on December 8 for a total of 2,541,199 cases, protective measures continued to be rolled back. While gloves and masks are required in the Moscow metro, citizens must register their telephone number before entering a bar or nightclub; moreover, museums are closed, international flights are gradually being reinstated, and students in first through fifth grade are returning to school [62]. As early as July, the mayor of Moscow announced that wearing masks would no longer be required outdoors as new COVID-19 cases dwindled in the capital [54]. Figure 1 shows the timeline of the COVID-19 pandemic from December 2019 to October 2020 in Central Asia.

Without an effective vaccine to prevent COVID-19, Central Asian leaders require an effective SARS-CoV-2 surveillance system that enables their governments to make safe and informed decisions [63-70]. Public health departments [71-76] plus several universities [77] and media outlets [78,79] are tracking the novel coronavirus using raw data regarding the number of new infections, testing, positivity, basic reproduction number (R_0_), and deaths, among other measures, such as local hospital capacity.

To that end, the objective of our research was to use a longitudinal trend analysis study design in concert with dynamic panel modeling and method of moments approaches to correct for existing surveillance data limitations [80,81]. Specifically, we measured significant weekly shifts in the increase, decrease, or plateaued transmission of SARS-CoV-2. Our study measured the underlying causal effect from the previous week that persisted through the current week, with a 7-day persistence rate to explain a clustering or declustering effect. The 7-day persistence represents an underlying disease transmission wave, where a large number of transmissions that resulted in a large number of infections on 1 day then “echoes” forward into a large number of new transmissions and hence a large number of new cases in the next 7 days. An example of the 7-day lag would be large sporting events in the United Kingdom that drew large crowds over an extended period of time even after cases were confirmed in the country. Other potential “super-spreader” events occurred in Turkmenistan, when a mass cycling rally was held on April 7 to celebrate World Health Day [82]. In summary, we measured negative and positive shifts in the transmission of SARS-CoV-2 or its acceleration and deceleration rates. We measured negative and positive shifts in the transmission of SARS-CoV-2 as well as the speed, acceleration or deceleration, and jerk rates along with the 7-day persistence, which do not suffer from sampling bias. For details, see Oehmke et al [80,81]. Our surveillance metric will provide public health surveillance data to inform governments that are making decisions regarding disease control, mitigation strategies, and reopening policies as they continue to manage this unprecedented situation.

## Methods

This study relies on a longitudinal trend analysis of data collected from the Foundation for Innovative New Diagnostics (FIND) [83]. FIND compiles data from multiple sources across individual websites, statistical reports, and press releases. Data for the most recent 8 weeks were accessed from a GitHub repository that compiles data from multiple sources on the web; data for the most recent 4 weeks were accessed from the GitHub repository [84]. This resulted in a panel of 14 countries in Central Asia with 47-50 days in each panel (N=696). An empirical difference equation was specified in which the number of new positive cases in each country at each day is a function of the prior number of cases, the level of testing, and weekly shift variables that measure whether the contagion was growing faster than, the same as, or slower than in the previous weeks. This resulted in a dynamic panel model that was estimated using the generalized method of moments approach by implementing the Arellano-Bond estimator in R (R Project) [85].

Arellano-Bond estimation of difference equations has several statistical advantages: (1) it enables statistical examination of the predictive ability of a model and the validity of the model specification; (2) it corrects for autocorrelation and heteroscedasticity; (3) it has good properties for handling data with a small number of time periods and large number of states; and (4) it corrects for omitted variables and provides a statistical test of correction validity. With these advantages, the method is applicable to ascertaining and statistically validating changes in the evolution of the pandemic within a period of ≤1 week, such as changes in the reproduction rate. See Oehmke et al [80,81] for a detailed discussion of the methods. Finally, we calculated these indicators to inform public health leaders of where to take corrective action at a local level. China enjoyed great success at controlling the pandemic by closing down smaller geographical regions, preserving the larger economy and preventing other adverse outcomes from a national quarantine.

## Results

### Country Regression Results

We analyzed the 12 countries that are included in the Central Asia region as defined by the World Bank. The results of the associated regression supporting the weekly surveillance metrics are captured in Table 1. The Wald statistic for regression was significant (χ^2^_7_=14,217, *P*<.001). The Sargan statistic for validity was insignificant (χ^2^_550_=9, *P*>.99) and failed to reject the validity of overidentifying restrictions.

As shown in Table 1, the 1-day lag coefficient is positive and significant (1.075, *P*<.001), suggesting a clustering effect where the number of cases on a given day impact the number of cases on adjoining days. The 7-day lag coefficient and the impact of the limited testing over the weekend on case counts (the “weekend effect”) are both insignificant. The shift parameters for the weeks of October 6 and 13 are also insignificant, suggesting that there were no major changes in the rate of disease transmission in the region between these 2 weeks in general. The coefficient for cumulative tests is insignificant.

**Table 1:** Arellano-Bond dynamic panel data modeling of the number of daily infections reported by country in Central Asia from September 30 to October 13, 2020.

Variable	Values	*P* value
1-day lag coefficient	1.075	<.001
7-day lag coefficient	–0.051	.89
Cumulative tests	0.000016	.55
Shift parameter, week of October 6	–0.157	.53
Shift parameter, week of October 13	–0.417	.12
Weekend effect^a^	–0.009	.99

^a^Weekend effect: impact of limited testing over the weekend on case counts.

### Interpretation: Central Asian Regression Results

The 7-day lag and shift parameters suggest that there have been no recent changes in disease transmission rates. Additionally, there is no weekend effect or cumulative test effect.

### Surveillance Results

Static and dynamic surveillance metrics for the weeks of October 6 and 13 are reflected in Tables 2-6, and Table 7 shows the most populous countries in Central Asia as of 2020. Tables 2 and 3 capture static metrics, including the number of new COVID-19 cases, number of cumulative COVID-19 cases, 7-day moving average of new cases, rate of infection, new deaths, cumulative deaths, 7-day moving average of number of deaths, and death rates. Novel dynamic metrics are reflected in Tables 4 and 5. These metrics include (1) speed, or the weekly average of new daily cases per 100,000 persons, (2) acceleration, or the day-to-day change in speed, (3) jerk, or the week-over-week change in acceleration, 4) 7-day persistence effect, or the number of new cases per 100,000 persons reported on a particular day that are associated with new cases reported 7 days previously. These novel metrics enable analysis of the impact of previous cases on current cases and identification of potential changes of the pandemic trajectory in the future.

Table 2 reflects static surveillance metrics for the week of September 30 to October 6, and Table 3 reflects those metrics for the week of October 7-13. The region averaged 11,730 new cases per day for the period ending on October 6 and 14,514 for the period ending on October 13. The infection rate increased across the region from 4.74 per 100,000 persons to 5.66. This increase in infection rate was accompanied by a slight increase in death rate from 0.09 per 100,000 persons to 0.11. Up to October 13, the region had reported 2,040,812 cumulative COVID-19 cases.

**Table 2:** Static surveillance metrics for the week of September 30 to October 6, 2020.

Country	New COVID-19 cases, n	Cumulative COVID-19 cases, n	7-day moving average of new cases	Infection rate (per 100,000 persons)	New deaths, n	Cumulative deaths, n	7-day moving average of the death rate	Death rate (per 100,000 persons)
Armenia	406	53,083	454.57	13.73	6	990	4.57	0.20
Azerbaijan	143	40,931	116.00	1.43	2	600	1.43	0.02
Cyprus	29	1876	19.00	2.42	1	23	0.14	0.08
Georgia	549	9245	482.71	14.76	4	58	3.14	0.11
Kazakhstan	66	108,362	64.86	0.36	21	1746	3.00	0.11
Kosovo	34	15,889	45.00	1.89	2	635	1.43	0.11
Kyrgyzstan	164	47,799	182.43	2.54	0	1066	0.29	0
North Macedonia	223	19,096	187.14	10.70	8	768	4.43	0.38
Russia	11481	1,231,277	9835.57	7.95	184	21,559	157.57	0.13
Tajikistan	40	10,014	41.14	0.43	0	78	0.43	0
Turkey	1511	327,557	1469.29	1.81	55	8553	60.43	0.07
Uzbekistan	397	59,343	427.00	1.18	4	489	3.29	0.01

**Table 3:** Static surveillance metrics for the week of October 7-13, 2020.

Country	New COVID-19 cases, n	Cumulative COVID-19 cases, n	7-day moving average of new cases	Infection rate (per 100,000 persons)	New deaths, n	Cumulative deaths, n	7-day moving average of the death rate	Death rate (per 100,000 persons)
Armenia	745	57,566	640.43	25.19	6	1032	6.00	0.20
Azerbaijan	277	42,381	207.14	2.76	3	612	1.71	0.03
Cyprus	83	2130	36.29	6.92	0	25	0.29	0.00
Georgia	569	12,841	513.71	15.29	9	102	6.29	0.24
Kazakhstan	83	108,984	88.86	0.45	22	1768	3.14	0.12
Kosovo	98	16,345	65.14	5.46	1	649	2.00	0.06
Kyrgyzstan	343	49,871	296.00	5.31	2	1092	3.71	0.03
North Macedonia	80	21,193	299.57	3.84	3	800	4.57	0.14
Russia	13,690	1,318,783	12,500.86	9.48	240	22,834	182.14	0.17
Tajikistan	37	10,297	40.43	0.40	0	79	0.14	0.00
Turkey	1632	338,779	1603.14	1.96	62	8957	57.71	0.07
Uzbekistan	323	61,642	328.43	0.96	2	511	3.14	0.01

**Table 4:** Novel surveillance metrics for the week of September 30 to October 6, 2020.

Country	Speed^a^	Acceleration^b^	Jerk^c^	7-day persistence effect on speed^d^
Armenia	15.4	0.4	0.3	–0.6
Azerbaijan	1.2	0.1	0	–0.1
Cyprus	1.6	0	–0.1	–0.1
Georgia	13.0	0.9	–0.2	–0.4
Kazakhstan	0.4	0	0	0
Kosovo	2.5	–0.2	–0.1	–0.1
Kyrgyzstan	2.8	0	–0.2	–0.1
North Macedonia	9.0	0.8	0.5	–0.3
Russia	6.8	0.3	0.1	–0.3
Tajikistan	0.4	0	0.0	0
Turkey	1.8	0	0.0	–0.1
Uzbekistan	1.3	–0.1	0.0	–0.1

^a^Speed: daily positives per 100,000 persons (weekly average of new daily cases per 100,000 persons).

^b^Acceleration: day-to-day change in the number of positives per day (weekly average per 100,000 persons).

^c^Jerk: week-over-week change in acceleration per 100,000 persons.

^d^7-day persistence effect on speed: number of new cases per day per 100,000 persons.

**Table 5:** Novel surveillance metrics for the week of October 7-13, 2020.

Country	Speed^a^	Acceleration^b^	Jerk^c^	7-day persistence effect on speed^d^
Armenia	21.7	1.6	0.7	–0.8
Azerbaijan	2.1	0.2	0.2	–0.1
Cyprus	3.0	0.6	0.4	–0.1
Georgia	13.8	0.1	0.5	–0.7
Kazakhstan	0.5	0	0	0
Kosovo	3.6	0.5	0.3	–0.1
Kyrgyzstan	4.6	0.4	0.2	–0.1
North Macedonia	14.4	–1.0	–1.6	–0.5
Russia	5.2	0.1	0.0	–0.2
Tajikistan	8.7	0.2	0.0	–0.4
Turkey	0.4	0	0.0	0
Uzbekistan	1.9	0	0.0	–0.1

^a^Speed: daily positives per 100,000 persons (weekly average of new daily cases per 100,000 persons).

^b^Acceleration: day-to-day change in the number of positives per day (weekly average per 100,000 persons).

^c^Jerk: week-over-week change in acceleration per 100,000 persons.

^d^7-day persistence effect on speed: number of new cases per day per 100,000 persons.

**Table 6:** Comparison of 1-day persistence in the four countries in Central Asia with positive significant positive accelerations for the week of October 6, 2020.

Country	1-day persistence	
	Week of September 30	Week of October 6
Armenia	16.1	21.5
Georgia	13.0	16.5
North Macedonia	8.8	14.8
Russia	7.0	9.1

**Table 7:** Most populous countries in Central Asia as of 2020.

Country	Population as of 2020, n
Russia	145,953,632
Turkey	84,621,255
Uzbekistan	33,469,203
Kazakhstan	18,776,707

Russia and Turkey had the highest 7-day moving averages in the region, at 9836 and 1469, respectively, for the week of October 6, and 12,501 and 1603, respectively, for the week of October 13 (Tables 2 and 3). In terms of infection rate, accounting for population, Russia was at 7.95 per 100,000 persons and Turkey was at 1.81 for the week of October 06. The countries that had the highest infection rates in the region included Georgia, at 14.76 per 100,000 persons, and Armenia, at 13.73. For the following week, the infection rate in Armenia jumped to the highest in the region, at 25.19 per 100,000 persons, while Georgia had a very slight increase to 15.29. Kazakhstan had the lowest infection rate in the region, at 0.36 per 100,000 persons for the week of October 6.

Russia and Turkey also had the highest 7-day moving averages of deaths in the region, with Russia at 157.57 per 100,000 persons for the week of October 6 and Turkey at 60.43. Together, they accounted for approximately 90% of the deaths reported in the region. Up to October 13, the region had reported 38,461 cumulative deaths. For the week of 10/06, North Macedonia and Armenia had the highest death rates per 100,000 persons in the region, at 0.38 and 0.20, respectively. Armenia maintained a death rate of 0.20 per 100,000 persons the following week. Georgia had the highest death rate the week of 10/13 at 0.24 per 100,000 persons, up from 0.11 the previous week.

The 1-day persistence is an indicator of a clustering effect where an event on a particular day causes an increase in the number of cases on adjoining days. As shown in Table 6, the 1-day persistence was highest in Armenia at 16.1, and it increased to 21.5 the following week. North Macedonia saw a large increase from 8.8 to 16.5, with Georgia and Russia seeing smaller increases from 13.0 to 14.8 and 7.0 to 9.1, respectively.

Largely consistent with infection rates, Armenia, Georgia, and North Macedonia had the highest speed or average of new daily cases per 100,000 persons. During the week of October 6, Armenia had a speed of 15.4, increasing to 21.7 the following week. Georgia had a speed of 13.0, which increased slightly the following week to 13.8. North Macedonia had a speed of 9.0, which increased to 14.4. The region overall had an increase in speed from 4.2 to 5.2.

**Figure 2:**
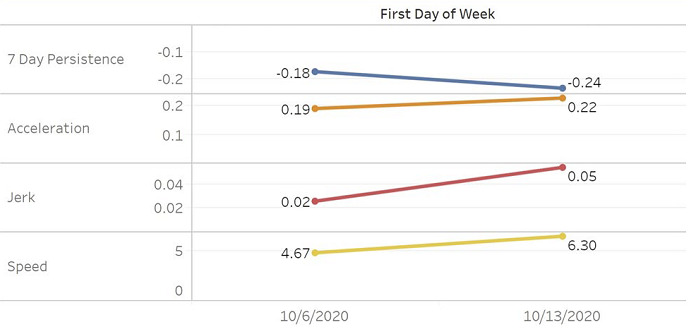
Weekly SARS-CoV2 trends in Central Asia [86].

Speed is best used in conjunction with acceleration and jerk, which can provide further insight into potential pandemic trajectory changes. Four countries in the region had significant positive accelerations for the week of October 6: Georgia at 0.9, North Macedonia at 0.8, Armenia at 0.4, and Russia at 0.3. North Macedonia, Armenia, and Russia also had positive jerks. During the following week, in addition to the highest speed, Armenia had the highest acceleration and jerk in the region. Figure 2 shows shows weekly trends of SARS-CoV-2 in Central Asia [86], and additional trends can be found in Multimedia Appendices 1-5.

## Discussion

### Principal Results

COVID-19 poses a significant threat to the Central Asian region, which is largely composed of former Soviet republics. These countries continue to suffer from food insecurity, high levels of poverty, and variation in health care quality and access as the region continues on its journey of transitioning from a centralized Soviet medical system. The population also suffers from multiple endemic infections, such as HIV/AIDS and tuberculosis. Russia and Turkey comprise the bulk of the population in Central Asia, and these countries are facing growing burdens of chronic disease and some of the highest obesity rates in Europe. Due to the combination of these factors, the region is vulnerable to negative outcomes from the COVID-19 pandemic. To date, the region has seen variations in policy intervention to control the spread of COVID-19 and mitigate outbreaks. Some countries, such as Kazakhstan and Azerbaijan, imposed strict and early lockdowns, while others, such as Russia, imposed more limited interventions.

Metrics tracking the progression of COVID-19 in Central Asia to date have largely been static, including measures such as new cases, cumulative cases, deaths, and 7-day moving averages. These metrics provide a view of the current state of the pandemic but are unable to provide any insight into the change in the speed of the pandemic over time or potential shifts in its trajectory, evolving from controlled spread to rapid growth or vice versa. These metrics also provide limited utility in comparing countries to each other and in analyzing countries with smaller populations. Novel metrics such as speed, acceleration, and jerk help contextualize static metrics and provide a view of trajectory over time, enabling potential anticipation of how the pandemic will evolve in the future.

Considering the static and dynamic metrics, it is apparent that the Central Asian region is trending negatively. The region saw an increase in 7-day moving average of new cases, infection rate, and speed for the week of October 13 compared to the week of October 6, with continued positive acceleration. This trend is largely driven by Russia and Turkey, which together encompass over 70% of the region’s population and showed the highest 7-day moving averages in the region. Russia has the fourth highest speed in the region and continues to experience positive acceleration.

Kazakhstan, the fourth most populous country in the region, had the lowest infection rate for the week of October 6. This is likely due to a continued emphasis on policy interventions to curb the spread of COVID-19. Authorities in Kazakhstan took some of the earliest precautions to prevent infections and continue to strictly enforce pandemic mitigation measures, including halting the easing of restrictions due to the global spike in COVID-19 cases in recent weeks.

Turkey, while contributing a significant portion of the total cases in the region due to its population size, has maintained relatively low infection rates of 1.81 and 1.96 per 100,000 persons for the weeks of October 6 and 13, respectively. Turkey has taken significant precautions to mitigate the spread of the virus, and authorities continue to enforce rigorous mask wearing and social distancing guidelines along with local quarantines when necessary.

Armenia is experiencing uncontrolled spread of COVID-19, with an infection rate of 13.73 per 100,000 persons for the week of October 6 quickly jumping to 25.19, the highest in the region, the following week. The pandemic speed, consistent with the infection rate trajectory, increased from 15.4 to 21.7, with an acceleration increase from 0.4 to 1.6. This change is likely due to the recent lifting of the COVID-19 state of emergency, which allowed the resumption of in-person schooling and international flights, among other activities.

After Armenia, Georgia and Russia had the highest infection rates in the region for the week of October 13. Russia continues to resist implementing interventions to curb the spread of COVID-19, with no mask mandates, capacity caps, or nightlife restrictions. In addition to the significant focus on developing an effective vaccine, there has been limited intervention to manage the spread of COVID-19 in Russia. This policy stance is impacting the trajectory of the region, which is trending negatively with no sign of a reversal in sight.

### Conclusion

The rapid evolution, novel outbreaks, and frequently fluctuating trajectory of COVID-19 cannot be adequately assessed using static public health measures alone. Static measures, including the number of new COVID-19 cases, number of cumulative COVID-19 cases, 7-day moving average of new cases, rate of infection, number of new deaths, number of cumulative deaths, 7-day moving average of number of deaths, and death rates, provide a current view of the state of the pandemic. However, these measures do not provide any insight into how the trajectory of the pandemic may change over time.

Generally, the approach to modeling the spread of COVID-19 is to assume there is an underlying contagion model [87] and then to attempt to measure those model parameters [88], which involves contact tracing to determine the spread of the disease [89-92]. With an incubation period of up to 14 days [93], modeling this spread can take months. Early estimates of COVID-19 were developed using the method by Lipsitch et al [94], which was used for contact tracing in Wuhan and Italy; however, the statistical properties were weak [95-100]. Zhao et al [96] estimated the serial interval distribution and R_0_ based on only 6 pairs of cases, which is insufficient to understand the transmission of COVID-19 [101]. This results in relaxing of the assumptions in these models, such as disaggregating the population by geography and modeling within-geography and across-geography personal interactions [102]. Martcheva [103] developed a dynamic model from several contagion models and their possible dynamics [104,105]. They are limited to the statistical inference of parameter values from actual data [106].

Novel surveillance metrics allow for a more nuanced analysis of the COVID-19 pandemic and together with static metrics, can enable policymakers to make informed decisions to control the spread of the pandemic and prevent further outbreaks. Novel dynamic metrics include speed, acceleration, jerk, and 7-day persistence, and they provide potential insight into how the pandemic will evolve in the future.

The analysis of Central Asia using static and novel surveillance metrics suggests that the region is precariously positioned and trending negatively. Russia, the largest country by population, continues to have high infection rates and one of the highest speeds of infection. With no sign of increasing restrictions, it is unlikely that this trend will reverse and that outbreaks in the region will be controlled.

### Limitations

Our data are limited by reporting methods across individual countries. Some countries, such as Turkmenistan, refuse to acknowledge COVID-19 cases. Variation in testing and infrastructure may impact the number of cases reported by other countries. The data are reported at a national level, which does not enable any subnational analysis.

### Comparison to Prior Work

This study is part of a broader research program at Northwestern Feinberg School of Medicine (The Global SARS-CoV-2 Surveillance Project: Policy, Persistence, & Transmission). Novel surveillance metrics, including speed, acceleration, jerk, and 7-day persistence, have been developed by this research program and are being applied to all global regions.

## Appendix

Multimedia Appendix 1 Weekly Cental Asia SARS-CoV-2 statistics by country.

Multimedia Appendix 2 Weekly Central Asia 7-day persistence map.

Multimedia Appendix 3 Weekly Central Asia statistics.

Multimedia Appendix 4 Weekly Central Asia jerk map.

Multimedia Appendix 5 Weekly Central Asia acceleration jerk map.

## Abbreviations

FIND: Foundation for Innovative New Diagnostics
R_0_: basic reproduction number
USSR: Union of Soviet Socialist Republics
