# Supplementary figures and images for: Surveillance Metrics of SARS-CoV-2 Transmission in Central Asia: Longitudinal Trend Analysis
Source: J Med Internet Res. 2021 Feb 3;23(2):e25799. doi: 10.2196/25799 (PMC7861038; doi:10.2196/25799)

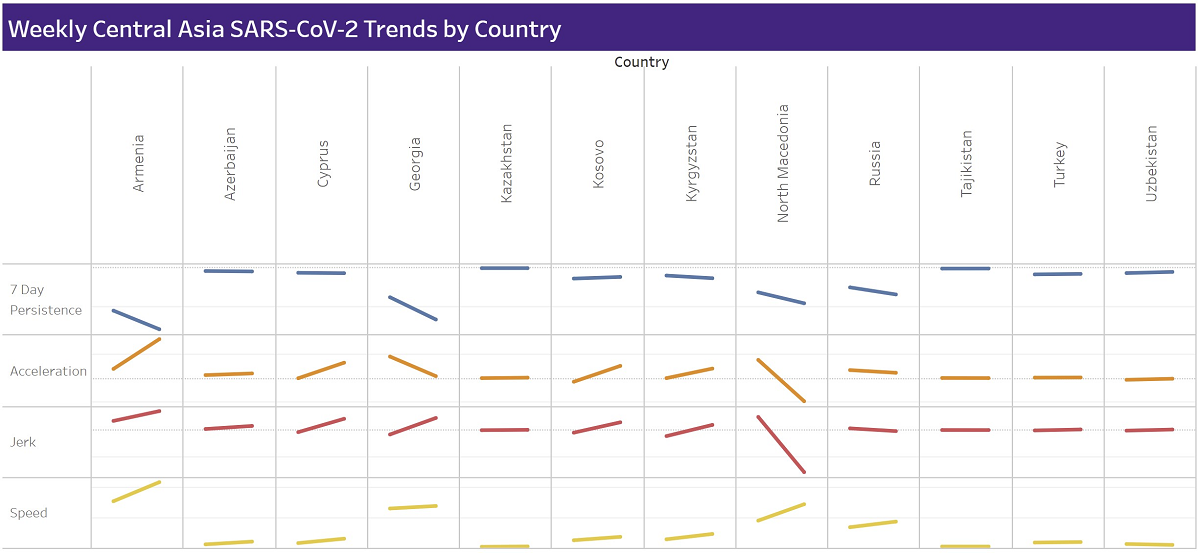

Supplement: Multimedia Appendix 1 [file jmir_v23i2e25799_app1.png]

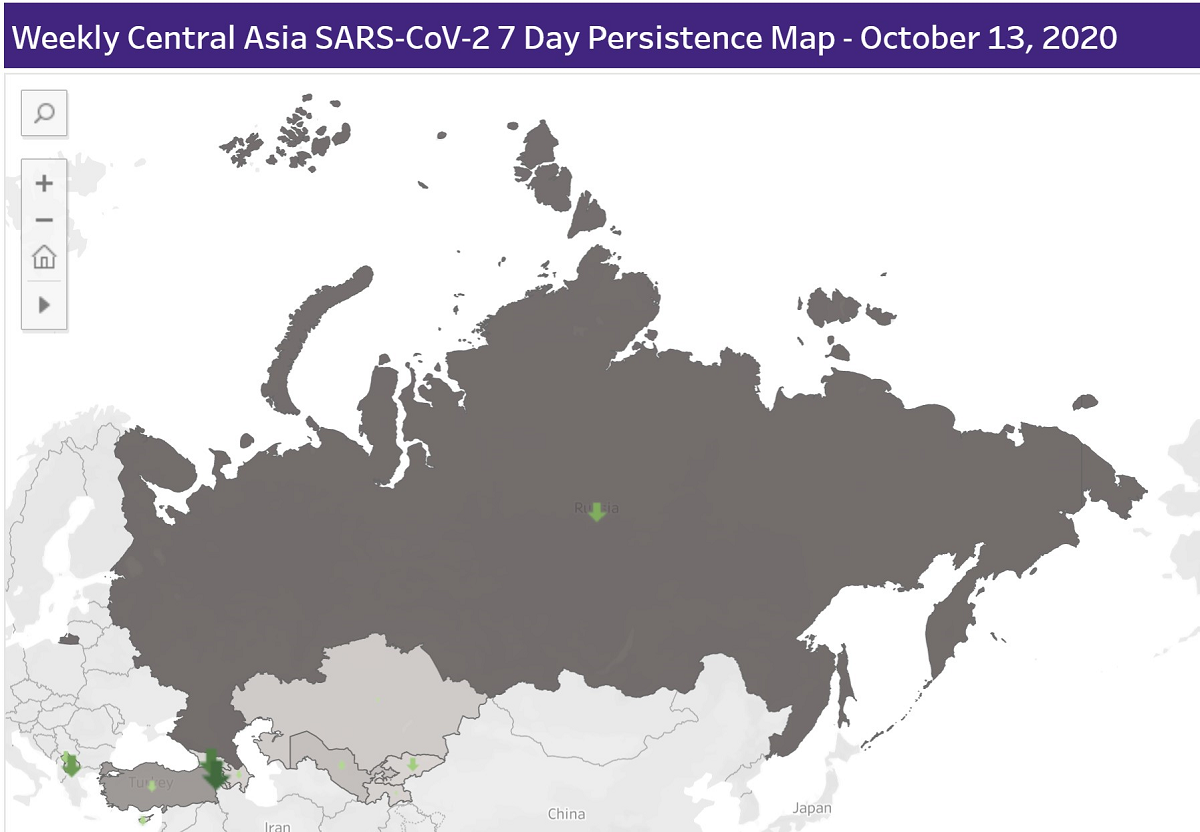

Supplement: Multimedia Appendix 2 [file jmir_v23i2e25799_app2.png]

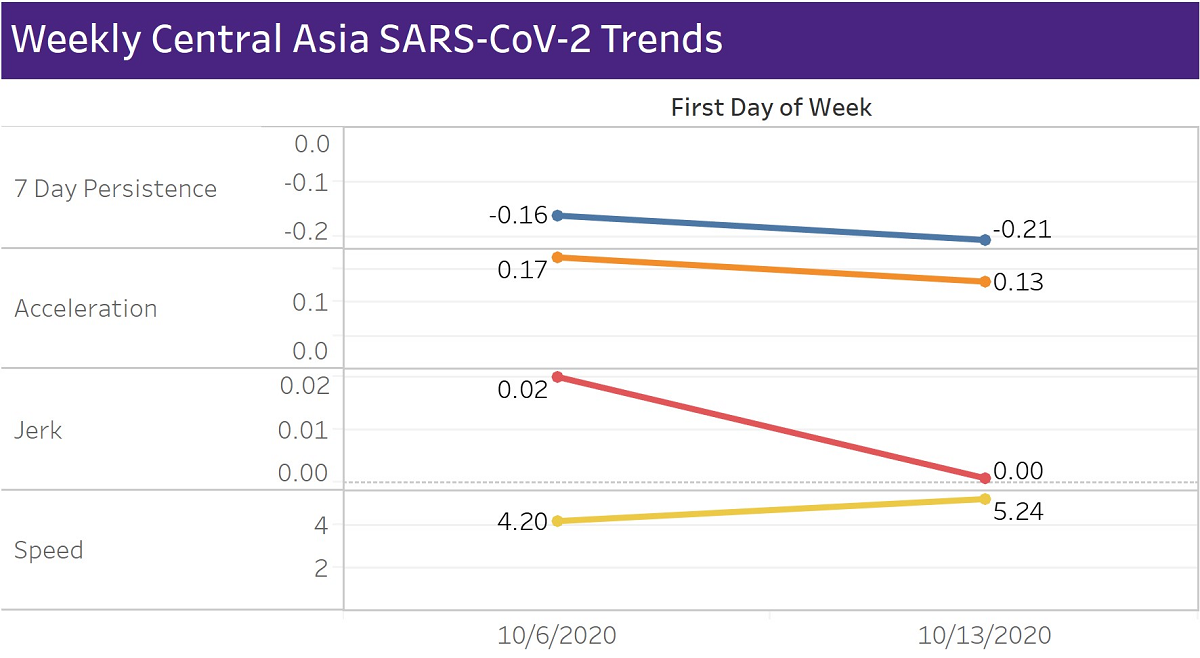

Supplement: Multimedia Appendix 3 [file jmir_v23i2e25799_app3.png]

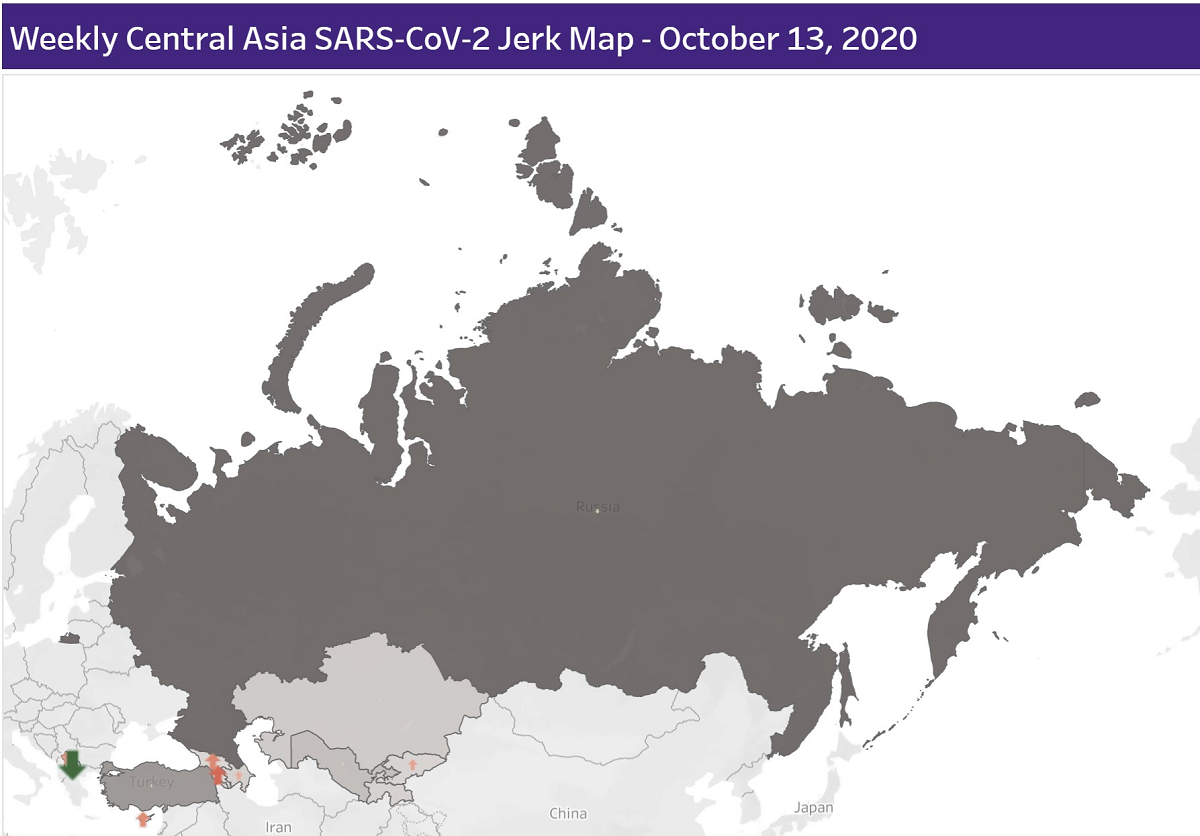

Supplement: Multimedia Appendix 4 [file jmir_v23i2e25799_app4.png]

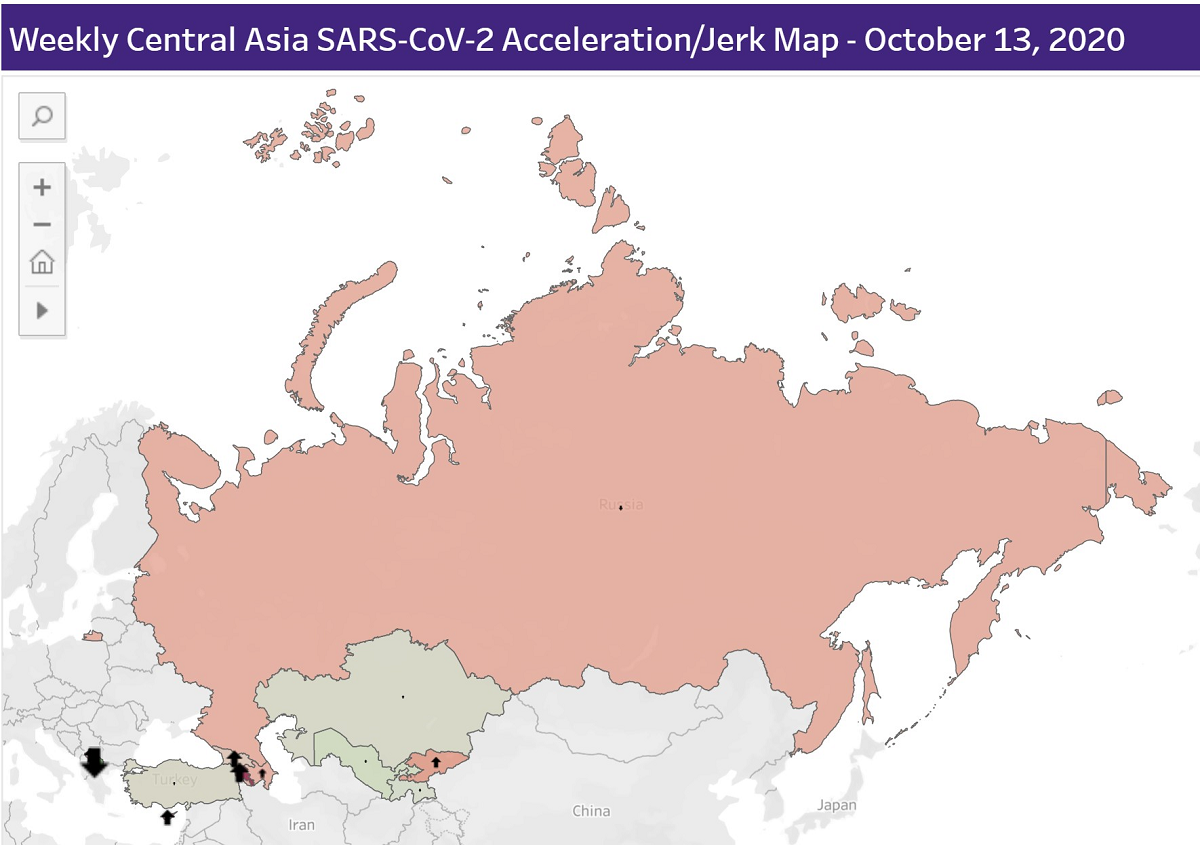

Supplement: Multimedia Appendix 5 [file jmir_v23i2e25799_app5.png]
